# Development of a Biodegradable Green Emitter Chitosan-Based OLED for Implantable Biomedical Devices

**DOI:** 10.1021/acsami.5c05499

**Published:** 2025-06-12

**Authors:** Filipa Pires, Eleonora Daini, Eleonora Vandini, Daniela Giuliani, Antonietta Vilella, Frederico Castelo Ferreira, Jorge Morgado

**Affiliations:** † Instituto de Telecomunicações, Instituto Superior Técnico, Universidade de Lisboa, Av. Rovisco Pais, Lisboa 1049-001, Portugal; ‡ Department of Biomedical Metabolic and Neural Sciences, 9306 University of Modena and Reggio Emilia, Via G. Campi 287, Modena 41125, Italy; § Department of Bioengineering and iBB - Institute of Bioengineering and Biosciences, Instituto Superior Técnico, Universidade de Lisboa, Av. Rovisco Pais, Lisboa 1049-001, Portugal; ∥ Associate Laboratory i4HB—Institute for Health and Bioeconomy, Instituto Superior Técnico, Universidade de Lisboa, Av. Rovisco Pais, Lisboa 1049-001, Portugal; ⊥ Department of Bioengineering, Instituto Superior Técnico, Universidade de Lisboa, Av. Rovisco Pais, Lisboa 1049-001, Portugal

**Keywords:** transient bioelectronics, OLEDs, biodegradability, light therapy, body-integrated devices

## Abstract

Light is a crucial tool in medicine for diagnosis and treatment. New light sources called organic light-emitting devices (OLEDs) have been integrated with various extracorporeal and implantable devices to sense or stimulate specific cellular responses. However, one of the current challenges remaining is to design a transient OLED that functions effectively and completely dissolves inside the human body when it is no longer needed, without affecting homeostasis. This work addresses this issue by creating the first transient chitosan-based OLED. The OLED is turned on at low voltages and emits a bright green light with a maximum luminance of 36 cd/m^2^. In vitro assays showed that all layers break down under physiological settings, and the biocompatibility was confirmed by in vivo tests. The chitosan-based OLED represents a breakthrough in transient electronics and advances implantable light-based biomedical applications.

## Graphical abstract

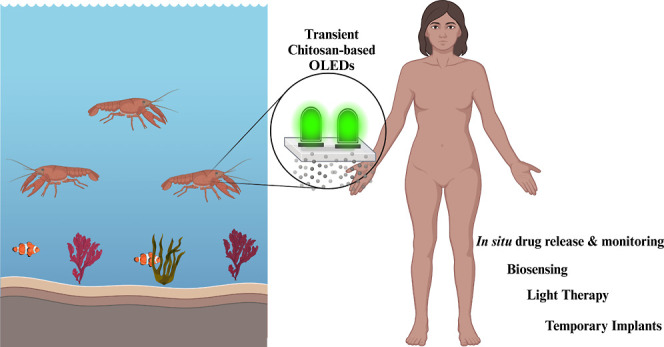

## 1 Introduction

In the past, the primary role of light on the human body was closely associated with managing of sleep patterns; however, it is now understood that light has numerous additional functions. For instance, light modulates cellular behavior and homeostasis by controlling the synthesis of essential vitamins, hormones, and cytokines that control the immune system, calcium metabolism, cognitive function, and glucose homeostasis.^1–3^

Since light has a major effect on regulating particular cellular processes, it has been clinically applied as a therapeutic agent/tool in contemporary medicine over the last few decades. For example, photodynamic therapy is an available therapeutic approach for cancer eradication that uses visible light as a tool to activate the photosensitizer (administered drug that specifically accumulates at the tumor site) to generate reactive oxygen species to intentionally destroy cancer cells by apoptosis, necrosis, or autophagy. This modality earned FDA’s approval and is currently used for treatment of certain skin disorders (like acne, basal cell carcinoma, psoriasis) and various kinds of cancer (such as head, neck, breast, lung, prostate, liver, gastric) and in the management of ulcer and chronic wounds.^4–7^

Another light-based method is photobiomodulation therapy in which specific cellular chromophores (like cytochrome *c* oxidase, opsins, and transient receptor potential ion channels) are stimulated by light at a specific wavelength and dose to trigger a particular cellular response (e.g., induce cell motility, proliferation, differentiation, apoptosis, necrosis, tissue regeneration, among others). Generally, red light (620–800 nm) is preferentially employed in most cases as it has the ability to penetrate deeper into muscles and human skin.^8–10^ Nonetheless, recent studies also found that green and blue light can serve as nonpharmacological adjuvants and, in certain cases, may even have a greater effect on cellular response than red light.^11–13^ Green light irradiation, for example, was used as a wireless strategy to modulate the membrane voltage of primary hippocampal neurons and blind retinal explants seeded onto poly­(3-hexylthiophene) (P3HT) films using the photoelectric effect. When exposed to green illumination, P3HT generates enough electrical current that depolarizes neurons and prompts them to generate action potentials that restore cell communication and photosensitivity in deteriorated retinas.^14,15^ P3HT and other biocompatible conjugated polymers (like PEDOT:PSS) have been exploited in the design of wire-free eye prosthesis to restore some degree of functionality to the visual system in individuals suffering from blindness or vision loss.^16–18^ Additionally, specific genes encoding light-sensitive proteins, such as channelrhodopsin-2 (ChR2, blue light), halorhodopsin (yellow light), and archaerhodopsin (green light), could be inserted into targeted neurons to precisely control their activity in response to light (optogenetics technique).^19–21^

Notwithstanding these promising results, a significant limitation to the widespread clinical use of this therapy is the inability of blue and green light to penetrate deep into internal tissues and organs within the human body.

Fortunately, a variety of implantable devices capable of guiding and delivering light directly to internal tissues have been shown to be effective in overcoming the absorption, reflection, and scattering phenomena (natural optical barriers) that normally restrict the passage of blue and green photons through the human body. For example, organic light-emitting devices (OLEDs)^22,23^ have been implanted in animals to serve as light sources to trigger a therapeutic response, as well as for real-time monitoring of certain metabolites and pharmaceutical drugs and in vivo imaging for diagnostic purposes. For instance, a flexible OLED fabricated on a parylene substrate inserted into the sciatic nerve of transgenic rats allowed muscle contraction when turned on. The results also showed that the OLED possesses adequate mechanical properties for in vivo implantation as it neither caused mechanical harm to tissues nor induced the immune system. Moreover, since the OLED was made of nonmagnetic materials, it allowed tracking brain activity using magnetic resonance imaging.^24^ In another study, a wirelessly powered red OLED was e-jet printed onto glass lenses as a phototherapeutic strategy to mitigate the oxidative stress induced by blue light exposure on retinal pigment epithelial cells, which, over time, disrupts photoreceptors and leads to progressive central vision loss. These findings indicated that the OLED emitting red light at 200 cd/m^2^ diminished free radical formation and, consequently, decreased the apoptotic death of cells.^25^

Significant progress has been made in the design of an OLED for implantable applications. However, most current designs incorporate materials like PEDOT:PSS and parylene, which are biocompatible but not biodegradable within the human body (though reports on PEDOT-based biodegradable devices do not always clearly state its non-biodegradability).^26–29^ Consequently, surgical removal once they have fulfilled their function is required, posing risks of infection and tissue damage. To overcome this limitation, the forefront of innovation now focuses on creating transient devices that efficiently perform their intended function within the human body and then naturally begin to dissolve or disintegrate once they are no longer needed without causing any harm.

This work embraced this challenge, with the primary goal of developing an OLED in which each layer (substrate, emissive layer, and electrodes) is both biocompatible and biodegradable under physiological conditions.

We note that a fully biodegradable (inorganic) LED was reported, but the emission spectrum was broad and of low intensity.^30^

In the last years, research on biodegradable OLED substrates has explored several polymers such as sodium alginate,^31,32^ cellulose,^33,34^ silk,^35^ poly­(vinyl alcohol) (PVA),^36^ and poly­(lactic-*co*-glycolic acid) (PLGA).^37,38^ While many of these polymers offer desirable properties such as transparency, chemical stability, flexibility, and biocompatibility, in some cases, such as cellulose and sodium alginate, their suitability for transient implantable OLEDs remains limited. The limitation is primarily due to the lack of consensus regarding their safety and the absence of specific human enzymes required for their degradation such as alginate lyases and cellulases. Consequently, significant uncertainties remain regarding how these polymers are processed within the human body, highlighting the critical importance and relevance of our findings.

Surprisingly, chitosan, a biopolymer with excellent transparency, high flexibility, and remarkable biocompatibility and biodegradability, has been underexplored in this domain. Chitosan is readily digested by the human body, as gastric juice and lysosomes from immune cells secrete enzymes such as chitinases and lysozyme that break the glycosidic bonds between *N*-acetylglucosamine units, thus forming oligosaccharides and glucosamine that are subsequently metabolized to produce energy.^39–41^ In light of this, this study investigates the potential of using chitosan as a substrate for fabricating transient OLEDs under physiological conditions.

AlQ_3_(tris­(8-hydroxyquinolinato)­aluminum) was the first electroluminescent small molecule used in efficient green OLED devices.^42^ Considering also recent discoveries showcasing the therapeutic benefits of green light,^43–46^ we have decided to fabricate the first fully biodegradable green OLED in physiological conditions using this particular emitting material.

One major achievement of this work is presenting evidence that chitosan is a suitable and biodegradable polymer that can be used as a substrate to support the manufacturing and operation of an OLED device. The results also demonstrate that magnesium (Mg) and molybdenum oxide (MoO_3_), materials that undergo degradation (oxidation and hydrolysis, respectively) in the human body, are excellent candidates for the device electrodes.^47–50^

The fully biodegradable OLED has the structure chitosan/MoO_3_/Mg/MoO_3_/AlQ_3_/Mg, and it shows a low turn-on voltage of ca. 4 V, displaying a bright green peak emission at 526 nm (as expected for AlQ_3_ emission) and exhibiting a maximum luminance of 36 cd/m^2^. In vitro experiments further demonstrate that all components of this OLED break down under physiological settings (chitosan through enzymatic degradation and the remaining layers primarily through hydrolysis processes), and biocompatibility was confirmed by in vivo experiments.

Some potential applications of this transient green-emitting OLED are its use as a spatial and temporal adjuvant in guiding healing or tissue regeneration or to act as a local light source to excite other green-light-absorbing specific drugs or photosensitive compounds, enabling more precise diagnostic and therapeutic interventions. In chemotherapy, for example, real-time monitoring of the distribution and concentration of the anticancer drug is crucial to enable personalized dosing and minimize systemic toxicity. Knowing this, a promising direction lies in the integration of this chitosan-based OLED with other transient systems (such as batteries and optical fibers) to create a multifunctional device capable of simultaneously emitting therapeutic green light, monitoring a patient’s response, and delivering medication in a controlled manner, creating a comprehensive, closed-loop treatment system.

## 2 Experimental Section

### 2.1 Purification of Chitosan

Chitosan with a degree of deacetylation of 75–85% (Sigma-Aldrich) was initially dissolved in a 1% v/v acetic acid solution, and then, a 10% m/v sodium hydroxide (NaOH) was added to induce reprecipitation.^51^ The formed precipitate was vacuum-filtered through a 45 μm membrane (filter paper 1300/80, Filter-Lab, Filters AOIA SA, Spain) and washed successively with different mixtures of methanol and water (70:30, 80:20 and 100:0). Afterward, the precipitate (1 g) was placed into an extraction thimble (25 mm × 80 mm, Whatman, UK) and inserted into a 50 mL Soxhlet apparatus for continuous extraction of impurities, using dichloromethane, *n*-hexane, and acetone.

### 2.2 Production of Chitosan Substrates

After purification, chitosan was dissolved (1.5% m/v) in a 1% v/v acetic acid solution (pH 4) at room temperature for 24 h. The chitosan substrates were prepared by a solvent casting method, following a previously established procedure.^52^ Specifically, chitosan solution was poured (150 μL/cm^2^) onto a glass Petri dish and left at 30 °C for 16 h, resulting in a flexible and transparent substrate with a thickness of 50 μm.

### 2.3 UV/Vis Spectroscopy

UV/vis spectra of films and solutions were recorded with a JASCO V-730 UV–visible spectrophotometer (JASCO, Tokyo, Japan).

### 2.4 AFM Analysis

The surface topography of the chitosan substrate was analyzed by atomic force microscopy (AFM) using a Nano Observer microscope from Concept Scientific Instruments (Les Ulis, France), operating in the tapping mode under ambient conditions. The tips used were silicon probes with a tip radius of <10 nm and a resonance frequency between 200 and 400 kHz (APPNano, model: ACT). The scan resolution was 512 × 512 pixels, and the images were processed using the Gwyddion (version 2.56) software.

### 2.5 Fabrication of Conventional and Chitosan-Based OLEDs

ITO-coated glass substrates (VisionTek Systems Ltd., Chester, UK), 12 × 12 mm^2^, patterned with hydrogen chloride, and plain glass substrates (12 × 12 mm^2^) were utilized to fabricate conventional and partially biodegradable OLEDs, respectively. Both types of substrates underwent a meticulous cleaning process involving sequential washing with a non-ionic detergent, deionized water, acetone, and isopropanol in an ultrasonic bath. Then, the substrates were dried using a nitrogen stream and subjected to UV-ozone treatment (PDC-002-CE, Herrick Plasma) for 3 min to enhance the hydrophilicity and ensure the uniformity of film deposition during the subsequent spin-coating and thermal evaporation processes. For the preparation of conventional OLEDs, a thin film of PEDOT:PSS (AI4083, Heraeus) was spin-coated onto the patterned ITO-coated glass substrates at a speed of 1800 rpm for 60 s, followed by annealing at 110 °C in air for 10 min. In the case of partially-biodegradable OLEDs, a thin bilayer consisting of Mg (20 nm) and MoO_3_ (20 nm) (as a hole injection layer, HIL, to enhance hole injection into the emissive layer) was thermally evaporated (MBRAUN, MB–20G) onto glass substrates inside the glovebox. For the biodegradable OLEDs fabricated on chitosan substrates, first, a 20 nm layer of MoO_3_ was deposited to planarize the surface and improve Mg adhesion. Subsequently, layers of Mg (anode material) and MoO_3_ were thermally evaporated following a procedure analogous to that employed for the partially biodegradable OLEDs.

The emissive layer in both types of OLEDs comprised a 60 nm thick film of tris­(8-hydroxyquinoline) aluminum­(III) (AlQ_3_, CAS: 444 561, Merck), which was deposited by thermal sublimation under a high vacuum (3 × 10^–6^ mbar). Following this, a cathode layer of magnesium (80 nm) was deposited onto the substrates by using a shadow mask to define an array of four pixels per substrate, with each pixel having an approximate area of 8 mm^2^.

All devices were fabricated inside a MBraun glovebox with an incorporated thermal evaporator.

### 2.6 OLED Characterization

The characteristic current–voltage and luminance–voltage relations for each fabricated OLED were obtained at room temperature under vacuum, using a K2400 Source Meter and a calibrated photodiode.^52^ The light emitted from each pixel, at a particular voltage, was collected by an optical fiber coupled to a calibrated CCD detector (ScanSci).

### 2.7 Electrical Characterization of the Magnesium Anode on Chitosan Substrates

The sheet resistance and electrical resistivity of magnesium evaporated on chitosan substrates were determined by using the four-contact probe method inside a MBraun glovebox. After evaporating a 10 nm layer of molybdenum oxide to planarize the chitosan surface and 10 nm of Mg, four equally spaced gold stripes (30 nm thick, spaced 300 μm apart) were evaporated on top of it. The resistance of each sample was determined by applying a current (Keithley 2400 source, Cleveland, OH, USA) through the outer gold stripes and measuring the voltage drop across the inner stripes. The sheet resistance (R_S_) was calculated using the following eq 1

1 $$R_{s}=\frac{R\times w}{l}$$

where *R* is the calculated resistance (voltage drop divided by the current), *w* is the width of the magnesium band, and *l* is the spacing between the gold contacts. The electrical resistivity (ρ) was then determined by multiplying the sheet resistance with the thickness of the magnesium film (10 nm in this case), disregarding a possible contribution of the underlying layer of the semiconductive MoO_3_, which is, indeed, much less conductive than magnesium.

Subsequently, the Haacke’s figure of merit (FoM)^53^ was calculated, taking into account transmittance at 520 nm (*T*) and sheet resistance (eq 2).

2 $$FoM=\frac{T^{10}}{R_{s}}$$

### 2.8 Biodegradability Assays

To evaluate the degradability of the emissive layer material in OLEDs under physiological conditions, a small amount of AlQ_3_ was dissolved in PBS solution at 37 °C, and the absorbance of the solution was monitored at specific time intervals using a Jasco V-30 spectrophotometer. Additionally, the biodegradable character of the chitosan-based OLEDs in their final configuration was also assessed under physiological conditions. Specifically, each OLED was immersed in 10 mL of PBS solution, in the presence or absence of lysozyme (1 mg/mL), and its absorbance was monitored over time.

### 2.9 In Vitro Cytotoxicity Assays

In this study, the cytotoxicity of the emissive material of the OLEDs was analyzed by preparing membranes composed of AlQ_3_ dispersed in DegraPol (ab medica, Italy). These membranes were prepared by dissolving adequate amounts of AlQ_3_ and DegraPol in chloroform. Two different weight ratios of AlQ_3_/DegraPol were used: 1.8 × 10^–2^ wt % (Membrane I) and 0.18 wt % (Membrane II). The solutions had a total of ca. 74.5 mg of solids per mL of solution. Ten milliliters of each solution was casted into Petri dishes with an area of 24 cm^2^. The membrane final thickness was ca. 200 μm. The composition of membrane I, prepared with the 1.8 × 10^–2^ AlQ_3_/DegraPol wt % solution, was established in order to have an amount of AlQ_3_ per membrane unit area (5.6 μg) similar to that of a 60 nm thick film used in the preparation of the OLEDs (6.0 μg). Membrane II has a 10 times higher mass of AlQ_3_ per unit area. Subsequently, each membrane was sterilized under ultraviolet radiation for 2 h before cell studies.

Cytotoxicity assays were conducted using L929 fibroblast cells in agreement with the ISO10993-5 standard.^54^ The L929 cells were cultured in low-glucose Dulbecco’s Modified Eagle Medium (DMEM) supplemented with 10% fetal bovine serum (FBS) and 1% antibiotic–antimycotic (Gibco, Grand Island, NY, USA). Cells were maintained at 37 °C, 5% CO_2_, and 21% O_2_ in a humidified incubator. For the indirect cytotoxicity tests, extraction media were prepared by immersing each membrane (material to extractant ratio ∼3 cm^2^ per mL) in serum-free DMEM at 37 °C for 48 h. L929 cells were seeded at a concentration of 7.5 × 10 ^4^cells/cm^2^ in a 24-well plate with DMEM and allowed to attach for 24 h before the addition of the extraction media. After the initial attachment, the cells were exposed to the extraction media for 24 and 72 h. Cell viability was assessed using the MTT assay (TOX-1 kit, Sigma-Aldrich), performed according to the manufacturer’s instructions. Briefly, a solution of 1 mg/mL MTT was added to each well, and the plates were incubated at 37 °C for 2 h, protected from light. Following incubation, the resulting purple formazan crystals were dissolved, and the absorbance was measured at a wavelength of 570 nm. L929 cells cultured in standard DMEM and those exposed to small pieces of latex gloves (toxic material) were used as negative and positive controls, respectively.

For direct contact cytotoxicity assays, membranes (area ∼2 cm^2^) were placed directly on the top of near confluent L929 cells in 12-well plates and incubated at 37 °C for 72 h. After incubation, cells were examined under an inverted fluorescence microscope to qualitatively assess confluence and evaluate whether a halo of inhibition had formed at the cell–material interface.

### 2.10 In Vivo and Ex-Vivo Cytotoxicity Assays

#### 2.10.1 Animals

Two month old (at the start of the experiment) CD1 male (*n* = 9) and female (*n* = 11) mice were used for in vivo and ex vivo studies. Mouse progenitors were purchased from Charles River Laboratories Italia s.r.l. (Calco-Milano, Italy). Mice were housed in a pathogen free facility and kept in conditioned rooms with stable temperature (21 ± 0.5 °C) and humidity (60%) on a 12 h light/dark cycle with food and water available ad libitum. All animal procedures were approved by the Committee on Animal Health and Care of the University of Modena and Reggio Emilia and conducted in accordance with National Institutes of Health guidelines [CEE Council 89 609; Italian DL 26/2014, authorization *n*° 979/2020/PR]. All efforts were made to minimize animal suffering and reduce the number of animals used in this study.

#### 2.10.2 Surgical Procedure

Mice were randomly assigned to the Sham group (*n* = 9), DegraPol group (mice implanted with a DegraPol membrane, *n* = 6), and DegraPol + AlQ_3_ group (mice implanted with membrane I with 1.8 × 10^–2^% of AlQ_3_, by weight, *n* = 5). Before surgery, mice were deeply anesthetized with 3% isoflurane and shaved in the back; then, the upper back skin was disinfected with betadine, lifted, and incised, and a subcutaneous pouch of 1.5 × 1.5 cm was created to insert the sample according to the experimental group. Mice from the sham group underwent surgery without having any sample implanted. The incision was sutured by applying a surgical stitch and treated with Neuflan gel (0.5 g neomycin/0.025 g fluocinolone acetonide/2.5 g lidocaine) to minimize local postoperative pain and the risk of infections.

#### 2.10.3 In Vivo Toxicity Evaluation

In order to exclude the toxicity of the DegraPol membrane and DegraPol + AlQ_3_ membrane I (with 1.8 × 10^–2^% of AlQ_3_, by weight), mice were monitored before and for 12 weeks after membrane implantation. Specifically, mice were subjected 1 and 2 weeks after implantation and then every 2 weeks to a modified SHIRPA protocol^55^ followed by behavioral assessment of motor coordination and spatial locomotion. Briefly, we monitored weight throughout the experimental period (3 months), and the weight of each animal was normalized to the weight the animal had prior to the implant. We also measured general health status parameters such as motor activity, coordination, presence/absence of tremor, lacrimation, eyelid closure, fur appearance, whisker movement, and defecation. Each parameter was individually scored (0 = absent parameters and 1 = present) to delineate the mouse phenotype.^56^ As concerns behavioral analysis, the mouse motor activity and exploration/anxiety in a new environment was measured by means of the open field (OF) test once a month for three months, as previously described.^55,56^ Briefly, after acclimatization in the experimental room (30 min), one mouse at a time was placed in the center of an open wooden chamber (50 × 50 × 40 cm^3^) with dark walls, and spontaneous behavior was recorded for 10 min. The open field arena was virtually subdivided into 3 zones, namely, periphery (within 10 cm of the walls), center (the rest of the arena), and corners (10 × 10 cm^2^), for assessment of anxiety. Traveled distance, maximum speed, and time spent in each zone were automatically recorded with the ANY-maze Video Tracking system (Stoelting). The percentage of time spent in the center of the arena was considered as an indirect index of reduced anxiety since mice tend to spend more time in the periphery. The apparatus was thoroughly wiped with 70% ethanol after each test to minimize olfactory cues. Behavioral test was performed by an operator unaware of the experimental group to avoid bias.

### 2.11 Ex Vivo Toxicity Evaluation

#### 2.11.1 Sacrifice and Blood and Organ Collection

After 3 months from surgery, under general anesthesia with inhaled isoflurane, blood was collected, allowed to coagulate at room temperature for 30 min, and then centrifuged at 1200 g for 15 min. Serum was isolated and kept at −20 °C until use. Organs (liver, spleen, kidney, heart, lungs, brain) were collected, macroscopically examined, and weighed; additionally, the skin on the implantation site was lifted and examined to detect macroscopic signs of local toxicity.

#### 2.11.2 Hepatic Toxicity Evaluation

Elevated levels of circulating liver enzymes such as alanine aminotransferase (ALT) are the index of hepatic damage. Serum ALT level was evaluated by means of an ALT activity assay according to manufacturer instructions (Sigma-Aldrich, *#*MAK052). Briefly, 14 μL of serum per mouse (in duplicate) was used, and after incubation and absorbance reading at 570 nm (Multiskan FC, Thermo Scientific), ALT activity (U/L) was calculated with a linear regression method, using a pyruvate standard curve.

#### 2.11.3 Statistical Analyses

All data are shown as mean ± standard error of the mean (SEM) and analyzed with univariate Analysis of Variance (ANOVA) or two-way repeated measures ANOVA, as appropriate, using SPSS software (version 26). The effect of sex was analyzed as a covariate. Significance was defined as *p* value (*p*) ≤ 0.05.

## 3 Results and Discussion

### 3.1 Biocompatibility and Biodegradability of AlQ_3_ in Physiological-Mimicking Conditions

One of the main criteria in designing transient implantable devices is ensuring both the biocompatibility and biodegradability of the materials used. Therefore, this study started by evaluating the suitability of AlQ_3_ as a bio-friendly emissive material for OLEDs. The results of these studies are illustrated in Figure 1A–C. A film (140 nm thick) of AlQ_3_ deposited on glass was exposed to PBS solutions to evaluate the degradation kinetics. The glass/AlQ_3_ substrate was placed at the bottom of a cuvette, which was filled with PBS (ca. 3.4 mL), closed (Figure S1A), and placed inside the cavity of the UV/vis spectrophotometer, and the absorption of the supernatant solution was measured over time. Figure 1A shows the evolution of the spectra over time, up to ca. 22 h. The spectrum is dominated by a narrow band centered at 240 nm, with a shoulder at 250 nm, attributed to π → π* transitions within the released quinoline ligands. This assignment is supported by the similarity with the absorption spectrum of 8-hydroxyquinoline in PBS (Figure 1B). The absorption spectrum of the AlQ_3_ film has a lower energy band, at ca. 393 nm, attributed to a metal-to-ligand transition, which is not detected in the supernatant solution, which leads us to conclude that such a transition is absent in the soluble materials, indicating the degradation (via hydrolysis) of the complex. Previous studies addressed the optical properties of AlQ_3_ in solution without degradation^57^ and have also addressed the degradation of AlQ_3_ upon hydration.^58^ The results of these two detailed studies are consistent with our findings. No fluorescence could be observed in the PBS supernatant solution upon exposure to UV light (254 nm). As shown in Figure 1A, the intensity of the 240 nm band grows over time, saturating after ca. 22 h, which we took as an indication that the entire AlQ_3_ of the film was degraded and solubilized. The film had an initial thickness of 140 nm coating the glass area of 0.88 × 0.85 cm, which translates into an AlQ_3_ mass of 10 μg, assuming a specific gravity of 1 for the AlQ_3_ film. Confirming evidence of total AlQ_3_ hydrolysis is provided by the fact that the glass that remained at the bottom of the cuvette showed no fluorescence, as shown in Figure S1B.

**Figure 1:**
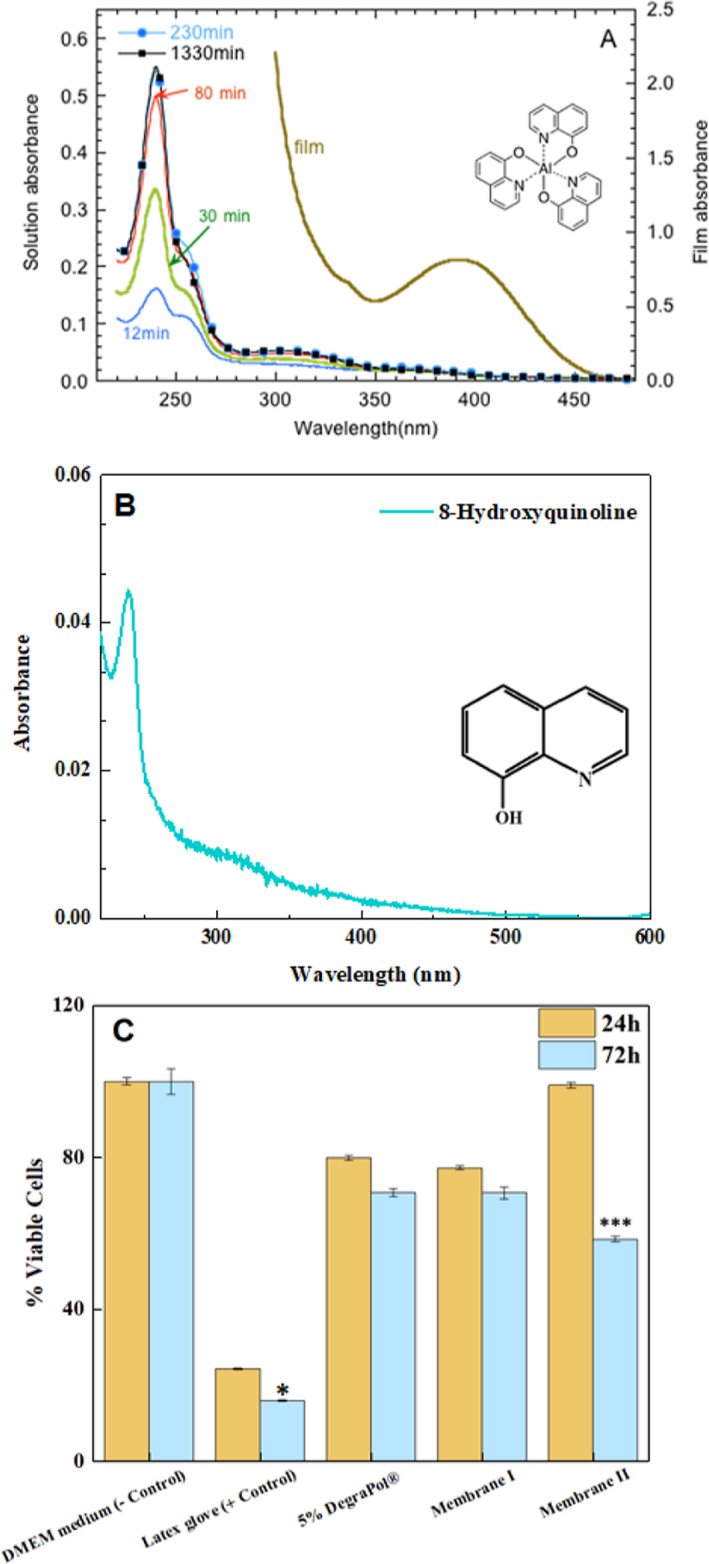
The emissive material AlQ_3_ used in our OLEDs undergoes hydrolysis in PBS solutions, producing nontoxic byproducts when a low concentration is used, while higher concentrations result in significant cell death. (A) Absorbance spectra illustrating the degradation of AlQ_3_ under physiological conditions over time, showing changes in key absorbance bands. (B) Absorbance spectrum of 8-hydroxyquinoline, the ligand of AlQ_3_, in PBS solution. (C) Cell viability results after incubation with lixiviates from DegraPol membranes containing two different concentrations of AlQ_3_ (1.8 × 10^–2^ wt % (Membrane I) and 0.18 wt % (Membrane II)) for 24 and 72 h, highlighting the cytotoxicity associated with higher AlQ_3_ content. The data are presented as the mean ± SEM from three independent experiments. In each experiment, each condition was assessed in triplicate. Asterisks (*) denote statistically significant differences (*p = 0.0264, ***p < 0.0001) between 72 and 24 h following cell incubation with lixiviates from the latex glove (positive control) and membrane II. No significant changes were observed for the other materials tested.

In a separate experiment, a film of AlQ_3_ on glass was immersed in PBS for 4 days. Its absorption spectrum was recorded before and after immersion. No evidence of remaining AlQ_3_ absorption was detected at the end (Figure S1C).

The results evidence a susceptibility of AlQ_3_ to hydrolysis, which is an essential requirement for its use in transient implantable devices. However, the biocompatibility of its degradation products must be thoroughly assessed as their interaction with cells could pose toxicity risks. Therefore, to evaluate whether the degradation products of AlQ_3_ are cytotoxic, we analyzed the toxicity of lixiviates from DegraPol membranes containing different AlQ_3_ concentrations (membrane I with 1.8 × 10^–2^% of AlQ_3_, by weight, and membrane II with a ten times higher concentration of AlQ_3_ (0.18 wt %)) after 48 h of immersion in PBS. The amount of AlQ_3_ in membrane I per unit surface area is similar to that of a 60 nm thick film of neat AlQ_3_. This approach aimed to determine if the hydrolytic byproducts of AlQ_3_ degradation represented a risk to cellular health. The results, displayed in Figure 1C, reveal distinct differences in cell viability depending on the concentration of AlQ_3_ in the membrane.

The lixiviates from membrane I resulted in cellular viabilities of 77% after 24 h and 71% after 72 h of post-incubation. These viability values are comparable to those observed for lixiviates of pure DegraPol membranes, a commercially available polyurethane polymer known for its biocompatibility. Cell viability above 70% over time aligns with the threshold generally considered acceptable for biocompatible materials (ISO 10993-5:2009),^54^ indicating that byproducts released from membrane I do not significantly impair cellular health over the tested time frame.

On contrast, the lixiviates from membranes with a 10 times higher AlQ_3_ concentration (membrane II) demonstrated a markedly different behavior. After 24 h, cell viability was approximately 99%, which is unusually high compared to that of the other samples. However, after 72 h, a significant decrease in viability was observed, indicating notable cell death likely caused by the accumulation of byproducts. Indeed, it is known that 8-hydroxyquinoline has chelating properties and interferes with cellular metal homeostasis.^59–61^ Hence, the amount of degradation products released from membrane II may exceed the threshold for cellular tolerance, resulting in the observed cytotoxicity after prolonged exposure.

In conclusion, these findings underscore the importance of carefully balancing the amount of AlQ_3_ incorporated into transient devices to minimize the release of potentially toxic byproducts, namely, 8-hydroxyquinoline. Functional OLEDs require a minimum thickness of about 60 nm, which means that the emissive area needs to be controlled, though the exposure of AlQ_3_ to the body fluids may take longer than in these assays as it will take time for capping layers to degrade and expose AlQ_3_, thereby reducing its degradation rate.

It is worth noting that in vitro cytotoxicity results do not always correlate with in vivo outcomes due to differences in the biological complexity and cellular environment interactions. To address this, we carried out additional in vivo biocompatibility assays to evaluate the safety and compatibility of membrane I under physiological conditions (Figure 2). These results demonstrate that, besides physiological sex-specific body weight gain over time (Figure 2A), no statistically significant difference was found following the implantation of DegraPol membranes, with (blue line) or without (red line) AlQ_3_ incorporation, throughout the whole experimental period (3 months). Interestingly, the mice showed a gradual weight gain over time, comparable to that of the control group (Sham), which did not undergo membrane implantation (Figure 2 A, gray line). Additionally, we quantified the alanine aminotransferase (ALT) activity in the serum 3 months after implantation as high circulating level of this enzyme is strictly correlated with alteration in liver function and hepatic toxicity. Figure 2B indicates that the hepatic enzymatic activity remained unaffected by the implantation as no significant increase of ALT activity was found in the serum of membrane-implanted mice with respect of Sham mice, thus suggesting the absence of hepatic damage as a result of DegraPol membranes implantation (with and without AlQ_3_). Similarly, the macroscopic appearance and weight of major organs, including the heart, liver, kidneys, and brain, were not significantly altered among experimental groups (Figure 2C). Moreover, the aspect of the skin on the implantation site was not altered, indicating the lack of major local or organ toxicity dependent on DegraPol membranes with and without AlQ_3_ (not shown). Furthermore, no alterations of the animal’s status, such as tremor, lacrimation, eyelid closure, fur bristling, baldness, and whisker immobility, were observed during the 3 month-long experimental period, in all experimental groups (data not shown).

**Figure 2:**
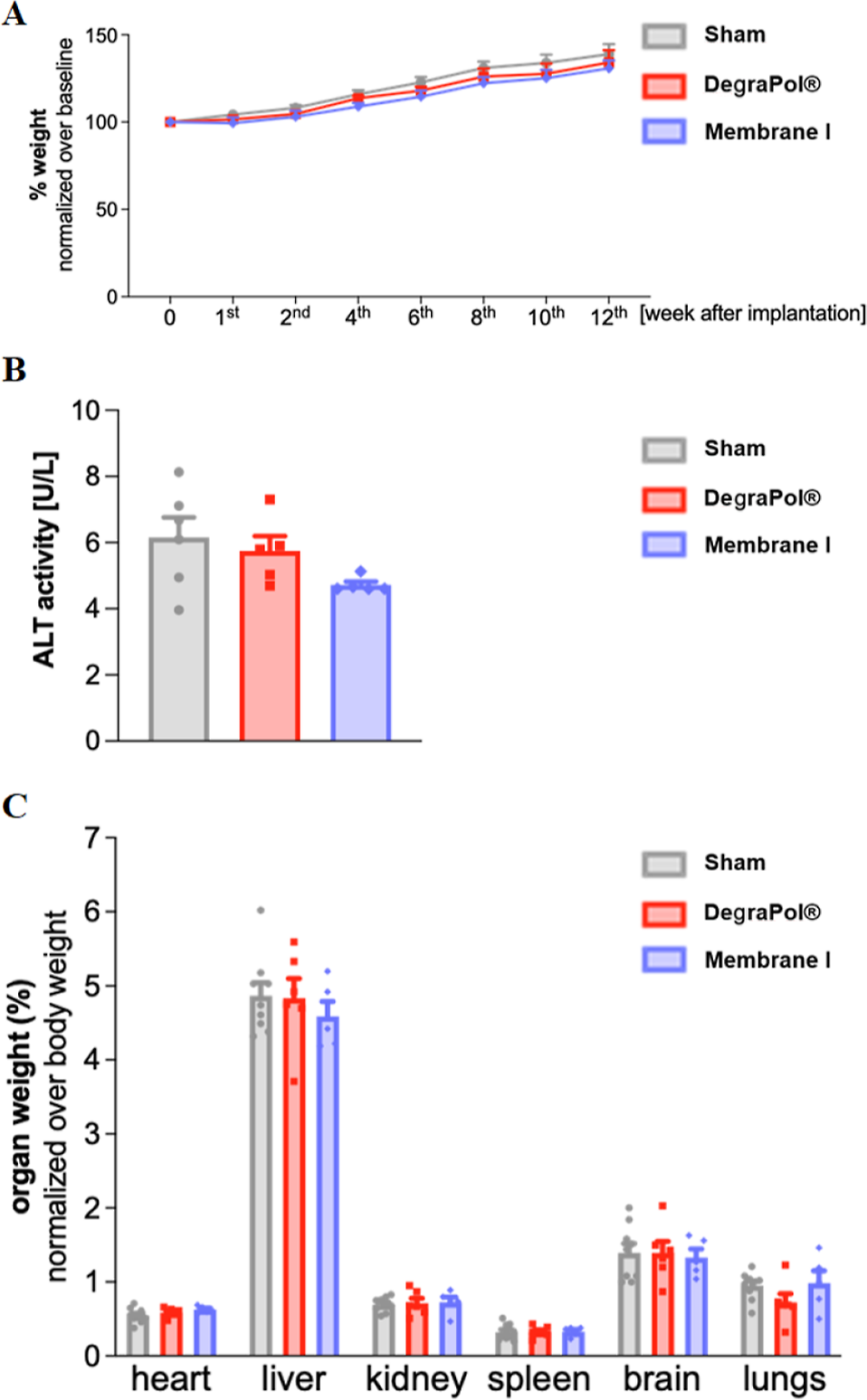
In vivo biocompatibility assays reveal no significant changes in body weight, organ appearance, organ weight, or ALT activity following implantation. (A) Weight gain expressed over body weight at the baseline (%) for sham (gray, *n* = 9) and mice implanted with DegraPol (red, *n* = 6) and membrane I (DegraPol with 1.8 × 10^–2^ wt % AlQ_3_ (violet, *n* = 5)) membranes from the 1st to the 12th week after implantation. Data are shown as mean ± SEM and analyzed according to repeated measures ANOVA, time and membrane type as factors and sex as covariate. Statistical report: Time F­(7,112) = 33.353, *p* < 0.001; membrane type F­(2,16) = 0.992, *p* = 0.392; time*membrane type F­(14,112) = 0.590, *p* = 0.868; sex F­(1,16) = 74.320, *p* < 0.001. (B) Analysis of alanine aminotransferase (ALT) activity of mice to evaluate hepatic function. Serum ALT activity (U/L) was determined in sham (gray, *n* = 6), DegraPol (red, *n* = 5), and membranes I (violet, *n* = 5) mouse groups. Data represented as mean ± SEM and analyzed according to univariate ANOVA, membrane type as an independent variable and sex as a covariate. Membrane type F­(2,15) = 2.221, *p* = 0.151; sex F­(1,15) = 0.000, *p* = 0.989. (C) Organ weight analysis postimplantation reveals no significant differences between the two groups compared to baseline measurements (sham mice without a membrane implanted), indicating a higher biocompatibility index. For each animal, organ weight was normalized over body weight (%). Data are shown as mean ± SEM (sham (gray, *n* = 9), DegraPol (red, *n* = 6), and membrane I (violet, *n* = 5) (mouse groups)) and analyzed according to repeated measure ANOVA, time and membrane type as factors and sex as a covariate. Organ type F­(5,80) = 100.397, *p* < 0.001; membrane type F­(2,16) = 0.137, *p* = 0.873; organ type*membrane type F­(10,80) = 0.990, *p* = 0.459; sex F­(1,16) = 0.836, *p* = 0.374.

Changes in spatial exploration and locomotion are precocious manifestations of malaise. Therefore, an open field test was performed before surgical procedures as the baseline and at 4, 8, and 12 weeks after implantation to assess alterations in the motor activity of mice. Obtained results demonstrate a significant variation in time of the traveled distance with the membrane type (Figure 3A). Instead, the time spent in the center of the arena (indirect index of exploration and anxiety: less time in this area means increased anxiety) and the maximum speed during motor performance were not significantly different in experimental mice groups (Figure 3B and C).

**Figure 3:**
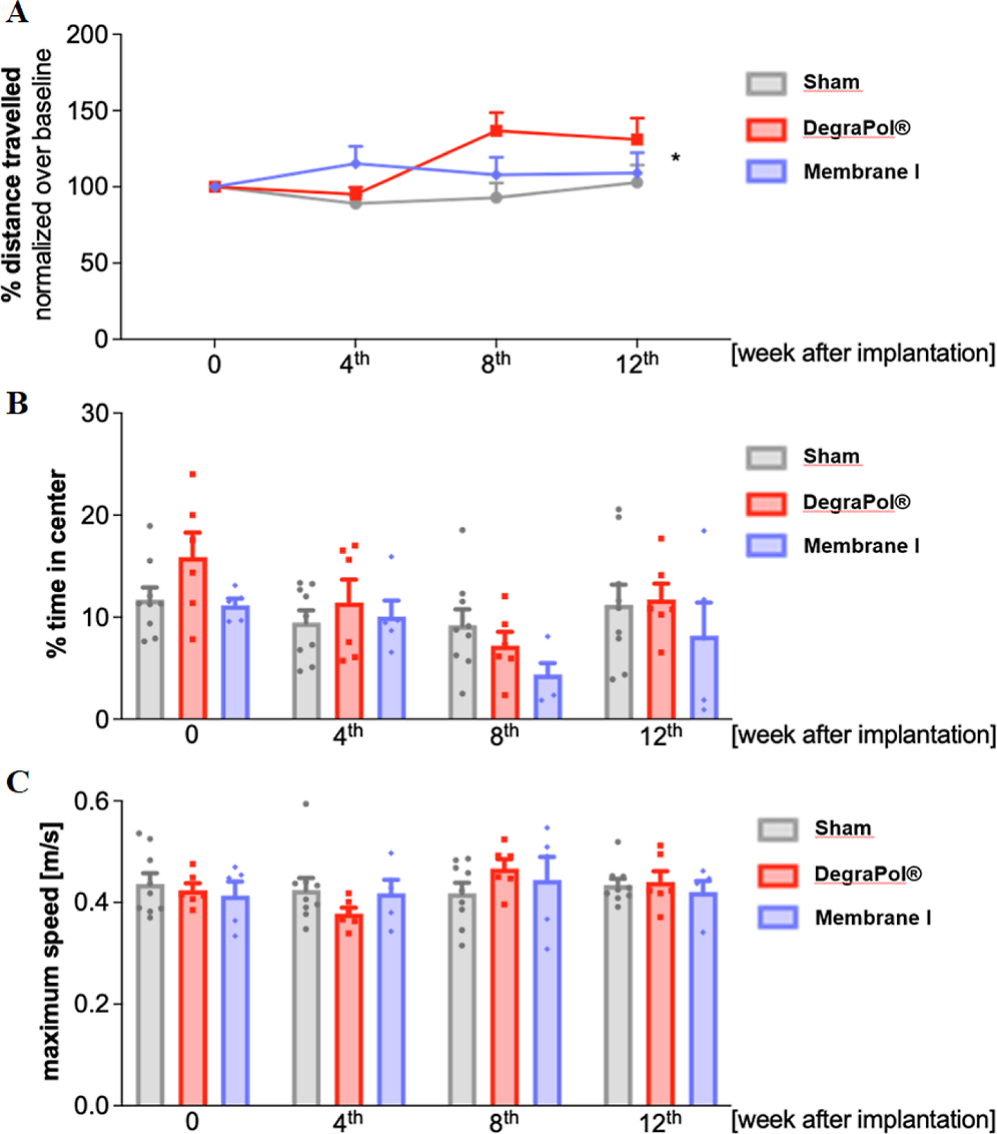
(A) Total distance traveled normalized over the baseline (%), (B) % of time spent in the center and (C) maximum speed in open field test performed at the baseline and 4, 8, and 12 weeks after implantation in sham (gray, *n* = 9), DegraPol (red, *n* = 6), and mice with implanted membranes I (DegraPol with 1.8 × 10^–2^ wt % AlQ_3_) (violet, *n* = 5) groups. Data are shown as mean ± SEM and analyzed according to repeated measures ANOVA, time and membrane type as factors and sex as a covariate. Statistical report: (A) Time F­(3,48) = 0.786, *p* = 0.508; membrane type F­(2,16) = 0.872, *p* = 0.437; time*membrane type F­(6,48) = 2.778, *p* = 0.021; sex F­(1,16) = 1.879, *p* = 0.189. (B) Time F­(3,48) = 2.180, *p* = 0.103; membrane type F­(2,16) = 1.398, *p* = 0.276; time*membrane type F­(6,48) = 1.129, *p* = 0.360; sex F­(1,16) = 0.029, *p* = 0.867. (C) F­(3,48) = 0.136, *p* = 0.938; membrane type F­(2,16) = 0.015, *p* = 0.985; time × membrane type F­(6,48) = 2.060, *p* = 0.076; sex F­(1,16) = 0.038, *p* = 0.847.

Summing up, these findings collectively highlight that the implantation of membrane I (DegraPol with 1.8 × 10^–2^ wt % AlQ_3_) does not trigger harmful side effects or compromise physiological stability or organ-specific functions. The material exhibits excellent biocompatibility, making it a promising candidate for use as an emissive layer in OLED technology, where safety and stability are paramount. As mentioned above, the AlQ_3_ film thickness was fixed at 60 nm, ensuring a good device performance and patient safety, maintaining its biocompatibility.

### 3.2 Material Selection for Developing Biodegradable OLEDs

As mentioned above, the most basic configuration of an OLED consists of a substrate (usually glass or plastic), an emissive layer (in this study, AlQ_3_), and two electrodes: the anode and the cathode. In most cases, indium–tin oxide (ITO), coating the substrate, is used as a transparent anode due to its high electrical conductivity and good stability, while PEDOT:PSS (poly­(3,4-ethylenedioxythiophene):poly­(styrenesulfonate)), with a work function of 5.1 eV, is employed as an additional layer to enhance charge (hole) injection and promote higher charge recombination efficiency in the emissive layer of the device. As electron injection electrode (cathode), usually low-work-function metals, such as calcium, barium, and magnesium, are used. This structure (designed here as a conventional OLED) can be modified to include additional layers to improve charge injection efficiency, electron-hole balance, and localization of the recombination zone.

Considering their unique optical, electrical, and mechanical properties, as well as their biocompatibility, both ITO and PEDOT:PSS have been integrated into various bioelectronic devices over the years. Some applications include neural interfaces, sensing electrodes, local drug delivery systems, and optogenetics. However, in the context of this study, their lack of biodegradability within the human body excludes them as possible candidates for the design of transient implantable OLEDs. ITO is brittle, and ITO debris is prone to migration and accumulation in lung tissue, potentially leading to pulmonary fibrosis.^62^ Alternative materials, including carbon black, single-walled carbon nanotubes, graphene and its derivatives (e.g., graphene oxide), and silver nanowires, have been investigated, but concerns persist about their degradation, potential for bioaccumulation, and adverse effects on human cells. Consequently, transition metals like zinc and other metal oxides (e.g., MoO_3_), along with alkaline earth metals such as Mg, currently represent the most suitable materials for transient devices due to their ability to dissolve harmlessly within the human body.

Mg was selected for the semi-transparent anode due to its high biocompatibility and biodegradable nature, thus serving as a substitute for ITO.^63,64^ It is important to note, however, that in terms of charge (hole) injection, Mg exhibits a much lower work function (3.7 eV) than ITO (4.5–5 eV depending on the surface treatment), which indicates that Mg is less effective in injecting holes. This can result in a charge imbalance in the emissive layer and, consequently, reduced device efficiency. To overcome this limitation, a thin film of MoO_3_, a biocompatible and biodegradable oxide, as mentioned above, with a reported work function of 6.8 eV,^65^ was deposited onto Mg to reduce the hole injection barrier to the adjacent organic layer of AlQ_3_. Mg was also used as the cathode material. The final active OLED structure is Mg­(semitransparent)/MoO_3_/AlQ_3_/Mg.

As shown in Figure S2, the transmittance of the Mg/MoO_3_ bilayer on glass is lower than that of ITO/PEDOT:PSS. At 520 nm, the transmittance of glass/Mg/MoO_3_ is only 32% vs 88% of glass/ITO/PEDOT:PSS. The thickness of Mg was optimized as 20 nm in terms of the compromise between significant electrical conductivity while showing an acceptable light transmission.

Glass and, less commonly, some thermoplastic polymers (such as poly­(ethylene terephthalate) or PET) have been the substrates of choice in the fabrication of regular/standard OLEDs. The main challenge in developing substrates for transient implantable OLEDs lies in identifying a material that meets both fabrication and performance requirements (such as transparency and mechanical stiffness) while also addressing the specific needs for interaction with the human body, including biocompatibility, biodegradability, and flexibility. This led us to investigate the use of chitosan.

Chitosan, a natural polysaccharide derived from the deacetylation of chitin, is easily found in the cell walls of fungi and the exoskeletons of insects and crustaceans.^66–69^ It is widely recognized for its remarkable biocompatibility and antimicrobial and anti-inflammatory activity, making it a cornerstone in the development of drug delivery systems and scaffolds for tissue engineering.^70–73^ Surprisingly, it has been scarcely investigated in the context of OLED fabrication but used in organic thin-film transistors.^52^

Chitosan is hygroscopic, and its surface usually has a significant roughness. We optimized the preparation process of the chitosan films, and the AFM analysis of their surface topography reveals a homogeneous and smooth surface, with an RMS roughness of 1.14 nm (Figure S3). As shown in Figure S4, chitosan substrates show a good transmittance (ca. 75% at 520 nm for a membrane with a thickness of ca. 50 μm).

Mg films are usually difficult to prepare by thermal evaporation due to the poor adhesion to substrates, as is the case of chitosan. We circumvented this difficulty by incorporating a planarization layer of MoO_3_, which not only smooths the surface of the chitosan substrate but also enables uniform adhesion of Mg as the anode (Figure 4A). Figure 4A also illustrates the flexibility of the chitosan substrate and the uniform adhesion of both magnesium and AlQ_3_, as evidenced by the uniform green emission of the central band of the device when exposed to ultraviolet light (Figure 4A, right). Figure 4B compares the performance of AlQ_3_-based OLEDs, all with the same cathode material (Mg) but with different substrates and anodes. The OLED glass/ITO/PEDOT:PSS/AlQ_3_/Mg, corresponding to a standard structure, exhibits a low turn-on voltage (here defined as the voltage at which a luminance of 0.01 cd/m^2^ is reached, of ca. 4 V) and the highest luminance value of ca. 1400 cd/m^2^ at 8 V (*L*_MAX_ = 2024 cd/m^2^ at 8.5 V), among the series. The OLED where ITO/PEDOT was replaced by Mg/MoO_3_ (structure glass/Mg/MoO_3_/AlQ_3_/Mg) shows a lowering of the turn-on voltage to ca. 3 V, but the maximum luminance (at 8 V, ca. 90 cd/m^2^) is more than 1 order of magnitude lower. A decrease in the current flowing through the OLED is also observed. These results show that Mg/MoO_3_ has a lower hole injection ability than ITO/PEDOT:PSS.

**Figure 4:**
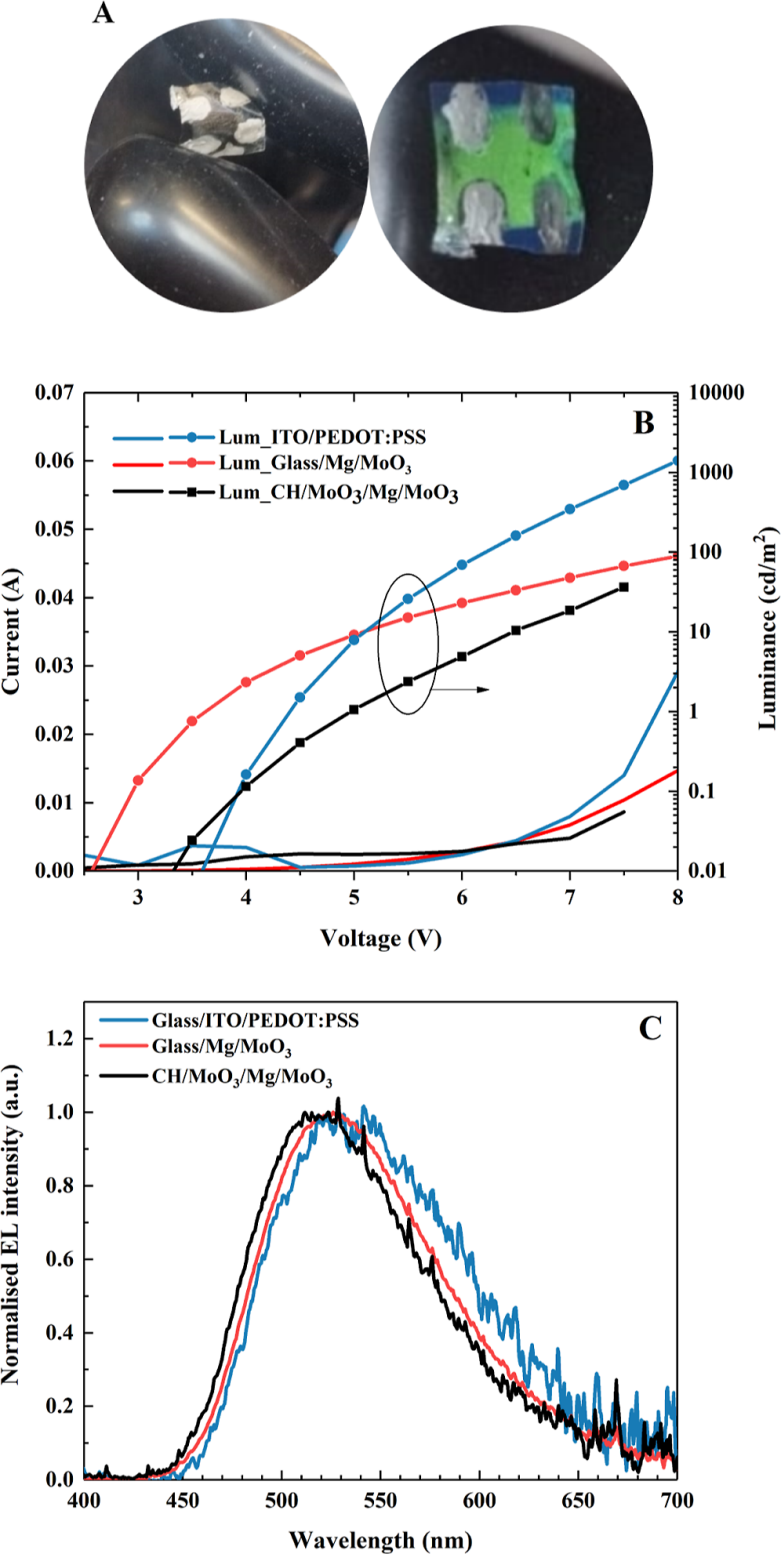
OLED performance variation when ITO/PEDOT:PSS is replaced by biocompatible and biodegradable materials Mg and MoO_3_. (A) Photos of the biodegradable OLED under ambient and UV light. The chitosan-based OLED has dimensions of 1.2 × 1.2 cm^2^, with a total thickness of ca. 50 μm (essentially the thickness of the chitosan substrate), and contains an array of four pixels per substrate, with each pixel having an approximate active area of 8 mm^2^. (B) Current and luminance outputs as a function of applied voltage for AlQ_3_-based OLEDs, where different substrates and anode materials are used. (C) Electroluminescence spectra of the OLEDs whose characteristics are shown in (B). The structure of the devices and the energy levels of the main materials are shown in Figure S5.

When chitosan is used as a substrate (chitosan/MoO_3_/Mg/MoO_3_/AlQ_3_/Mg), the turn-on voltage increases to ca. 3.5 V and the maximum luminance at 7.5 V is ca. 36 cd/m^2^ (at 8 V, the OLED degrades).

These results show that the use of the fully biodegradable structure leads to a significant loss in terms of luminance, along with a significant decrease of maximum electroluminescence efficiency, when comparing with the standard structure (0.03 and 0.4 cd/A, respectively) (Figure S6). The limited transmittance of both the semitransparent Mg (compared with ITO) and of chitosan (compared with glass) are key parameters affecting that performance (Figures S2 and S4). The evaporation of Mg on chitosan substrates significantly reduces their transparency, decreasing the transmittance at 520 nm from 75% to 41% after anode formation (Figure S4). The Mg anode on chitosan/MoO_3_ exhibits a sheet resistance of 85 ± 2 Ω/sq, corresponding to an electrical resistivity of 8.5 × 10^–5^ Ω·cm. Furthermore, we evaluated the performance of the Mg-based anode using Haacke’s figure of merit (FoM),^53^ achieving a value of 1.6 × 10^–6^ Ω^–1^, which falls within the acceptable range, particularly for emerging body-transient OLED applications. Although the FoM of 1.6 × 10^–6^ Ω^–1^ is significantly lower than that of ITO,^74^ which typically reaches 1.5 × 10^–2^ Ω^–1^, it still supports acceptable OLED operation. In fact, reasonable values of maximum luminance and efficiency are reached with this biodegradable structure.

The EL spectra shown in Figure 4C are similar for all tested OLEDs, as expected and typical of AlQ_3_ emission. The maximum occurs at ca. 530 nm for the standard structure, with a small blue shift when Mg is used as an anode, which we attribute to the decrease of Mg transmission when the wavelength increases (see Figure S2).

Despite the loss in performance when compared with the standard OLED structure, the performance of the fully biodegradable and biocompatible OLED is still remarkable.

Though the hole injection ability of MoO_3_ is lower than that of PEDOT:PSS, MoO_3_ remains one of the best biodegradable alternatives currently available for the hole injection layer (HIL) of an OLED. Moreover, in certain biomedical applications, such as low-intensity light therapy and localized drug delivery systems, a high luminance is not a primary requirement.

As mentioned earlier, Mg readily dissolves under physiological conditions. MoO_3_ dissolves and is naturally metabolized or excreted in the human body, thus offering a critical advantage for transient implantable OLEDs.

Oral supplementation with Mg may alleviate chronic migraines, depression, heart problems, and asthma, while MoO_3_ intake prevents anemia and dental caries. In particular, the American Food and Nutrition Board recommends a daily intake of 420 mg of Mg for men and 320 mg for women aged 31–70 years, along with 2 mg of MoO_3_ for adults.^75–77^ It is important to note that in this study, the amounts of Mg and MoO_3_ used were significantly lower than the recommended daily doses in order to avoid any potential adverse effects on patient health.

The major milestone of this work was the development of a transient OLED for biomedical applications. Hence, the biodegradability of the full device, with the structure chitosan/MoO_3_/Mg/MoO_3_/Mg/AlQ_3_/Mg, was systematically analyzed. While it is well-documented (as mentioned above) that Mg and MoO_3_ undergo oxidation and hydrolysis, respectively, within the human body, there is relatively limited information regarding the safety of hydrolysis-derived subproducts of AlQ_3_. As demonstrated in this work, the hydrolysis of AlQ_3_ produces subproducts that appear to be nontoxic to human cells under the tested conditions, alleviating potential safety concerns for biomedical applications.

The final stage of the study involved monitoring the degradation of the complete OLED device with a particular focus on whether the chitosan substrate would dissolve under physiological conditions. For that, the fully assembled chitosan-based OLED device was immersed in PBS solution, in both the presence and absence of lysozyme. Lysozyme is an enzyme present in various human tissues (such as liver and kidneys) and in certain bodily fluids including tears, saliva, and mucus, and it was chosen for this work because it is known to catalyze the hydrolysis of β (1 → 4)-linkages in polymers composed of *N*-acetylglucosamine and glucosamine units analogous to the chitosan backbone.

The chitosan membrane absorbs above 305 nm (Figure S4), which partially overlaps with the absorbance bands of hydrolysis byproducts of AlQ_3_. In the absence of lysozyme, degradation of the chitosan-based OLED occurs primarily through slow hydrolysis in the PBS solution. In fact, the results shown in Figure 5A indicate an increase in absorbance of the PBS solution in contact with the OLED, with bands centered around 240 and 300 nm, corresponding to the release of hydrolysis byproducts of AlQ_3_ into the solution. Clearly, the photographs in Figure 5B and Movie S1 reveal that the device tends to curl when immersed in PBS, and even after 24 h, while some degradation is evident, a substantial portion of the device remains intact. This suggests that nonenzymatic hydrolysis alone is insufficient for complete dissolution within the observed time frame. In contrast, the presence of lysozyme significantly accelerates the degradation process, as evidenced by the photographs in Figure 5B and Movie S2, which show that the device completely dissolves within 24 h. Additionally, absorbance measurements of the PBS solution with lysozyme indicate a notable decrease in the typical bands associated with lysozyme, suggesting that the enzyme is actively consumed during the hydrolysis of the β (1 → 4)-linkages in the chitosan backbone (Figure S7).

**Figure 5:**
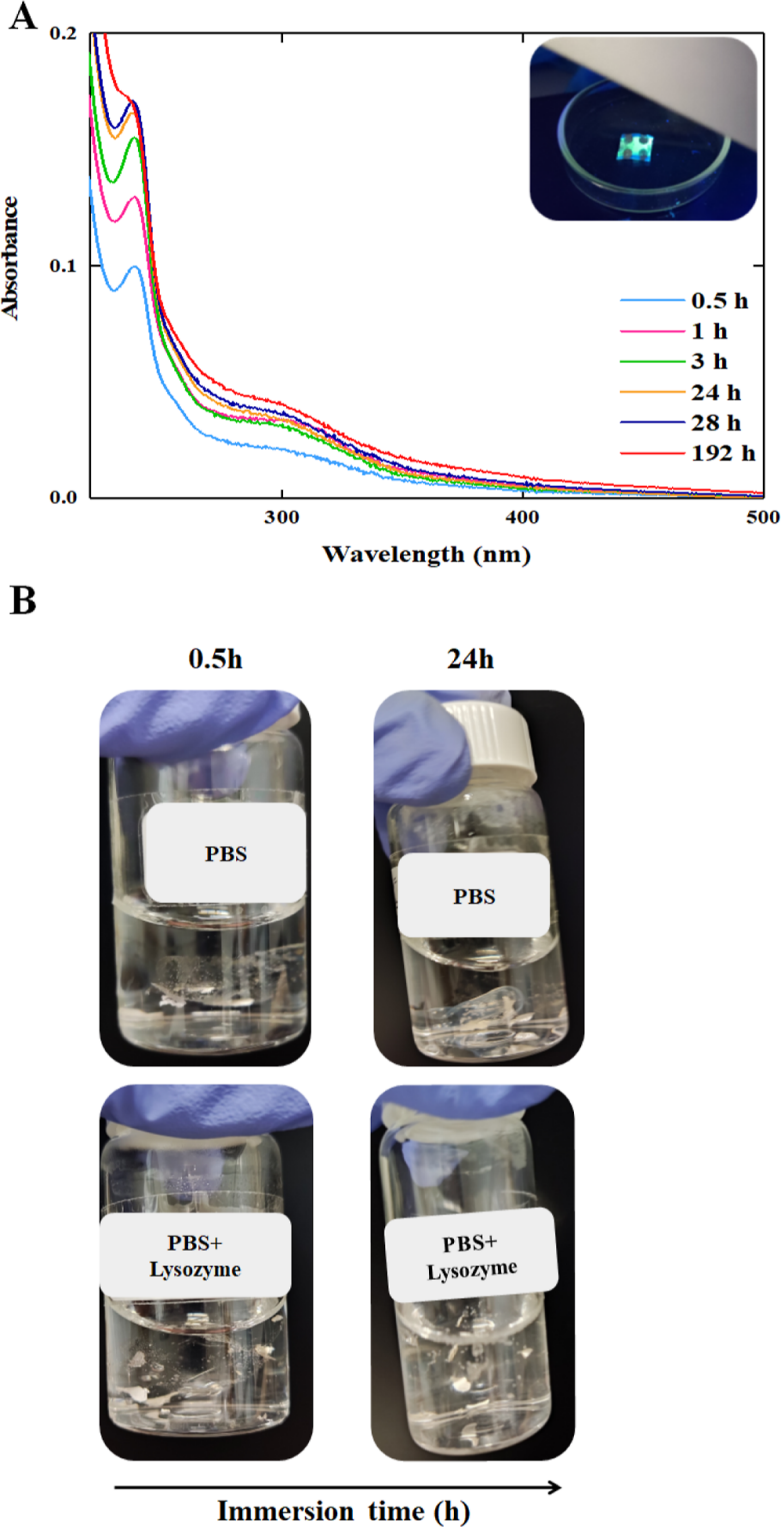
Degradation of the chitosan-based OLED in physiological mimicking conditions. (A) Absorbance measurements show the degradation of a chitosan-based OLED immersed in a PBS solution at 37 °C over a period of time. (B) The photographs demonstrate the degradation of the device, showing that after 24 h, the chitosan-based OLED had vanished entirely in the presence of the lysozyme.

In conclusion, the complete degradation of the chitosan-based OLED within 24 h in the presence of lysozyme is a major result, demonstrating the transient nature of the developed device. Nonetheless, for therapeutic applications requiring a time scale extending beyond a single day, this rapid degradation rate can pose a significant concern. In this study, as a proof of concept, a high concentration of lysozyme (1 mg/mL) was used, which is a hundredfold greater than the concentration present in human serum (10 mg/L).^78^ Consequently, under in vivo physiological conditions, it is anticipated that the degradation rate of the OLED would exceed 24 h due to the substantially lower availability of lysozyme. Even so, a strategy to prolong the lifetime of this device in physiological conditions relies on its encapsulation using a biodegradable polymer PLGA. The results presented in Figure S8 demonstrate that PLGA works as an effective diffusion barrier against both oxygen and moisture. The PLGA encapsulation ensures the stability of magnesium-based electrodes for up to five months when stored outside of an inert atmosphere, thus meaning that it can facilitate the deployment of these OLEDs in implantable devices intended for extended medical use.

## 4 Conclusion

This study introduces the first chitosan-based OLED, chitosan/MoO_3_/Mg/MoO_3_/AlQ_3_/Mg, that achieves a luminance of approximately 36 cd/m^2^ at 7.5 V and emits bright green light.

A key innovation of this work is the use of Mg/MoO_3_ as the anode and hole injection layers, replacing the commonly employed ITO and PEDOT:PSS found in numerous reported OLED systems. While the ITO/PEDOT:PSS combination is highly effective in facilitating charge injection into the emissive layer, better than Mg/MoO_3_, these materials present significant challenges for transient long-term implantable devices as they are nondegradable.

In this study, we also focused on the biocompatibility and biodegradability of the emissive layer (AlQ_3_) of OLED. Our findings revealed that, in vitro, AlQ_3_ undergoes hydrolysis in aqueous environments, and its biocompatibility, as well as the toxicity of its byproducts, is concentration-dependent. For the amounts of AlQ_3_ used in the OLEDs, we found that the byproducts of AlQ_3_ hydrolysis did not induce cell death. Additionally, in vivo experiments confirmed that AlQ_3_ is well tolerated by the body (in the amounts used in the OLEDs) as it did not promote animal (mice) health alterations or changes in body weight, organ weight and appearance, and hepatic enzymatic activity.

This study also shows that the device degrades under physiological conditions within 24 h, which could be useful for providing an initial boost in applications such as the healing process of burns. Moreover, as proof of concept, our findings demonstrated that PLGA is a suitable biodegradable polymer for encapsulating this device, conferring it resistance to oxygen and moisture, enabling the magnesium-based electrodes to remain stable for up to five months when stored outside of a glovebox. This indicates that PLGA encapsulation will significantly extend operational lifetime of an OLED for in vivo long-term therapeutic purposes.

Summing up, our findings show that the developed transient OLED has the potential to open new therapeutic avenues as it provides a means of delivering light with specific wavelengths, green light in this case, which has been associated with various health benefits. Henceforth, the progress in this area will benefit from the investigation of other OLED structures, emitting at other wavelengths and with improved performance, in particular by developing anodes with higher hole injection efficiency as the hole injection from Mg/MoO_3_ is the limiting factor of the performance of the OLED here developed. A promising future direction involves the integration of bioresorbable power sources, such as biodegradable batteries or wireless energy harvesting circuits,^79,80^ with these chitosan-based OLEDs. This synergy will represent a significant step toward the next generation of transient implantable devices by enabling intelligent and self-powered systems that safely dissolve after serving their purpose, as well as avoiding follow-up surgeries to remove the implants.

## Supplementary Material







## Data Availability

All data needed to evaluate the conclusions in the paper are present in the paper and/or the Supporting Information.

## Notes

Additional experimental data, including the setup scheme used to monitor the degradation of the AlQ_3_ film upon contact with PBS, transmittance spectra, and the structure of the designed OLEDs (PDF)
After 24 h of immersion, the degradation of the chitosan-based OLED in PBS is limited as the polymer curled and exhibited poor dissolution (MP4)
In the presence of lysozyme, the chitosan-based OLED undergoes complete dissolution within 24 h, driven by the enzymatic hydrolysis (MP4)
