# Supplementary material for: Development of a Biodegradable Green Emitter Chitosan-Based OLED for Implantable Biomedical Devices
Source: ACS Appl Mater Interfaces. 2025 Jun 12;17(26):37510–22. doi: 10.1021/acsami.5c05499 (PMC12232276; doi:10.1021/acsami.5c05499)
Supplement: Supplementary file 1 [file am5c05499_si_001.pdf]

Supporting Information

Development of a biodegradable green emitter chitosan-based OLED for implantable biomedical devices

Filipa Pires,[*,†] Eleonora Daini,[‡] Eleonora Vandini,[‡] Daniela Giuliani,[‡] Antonietta Vilella,[‡] Frederico Castelo Ferreira[¶,§] and Jorge Morgado[†,‖]

[†]Instituto de Telecomunicações, Instituto Superior Técnico, Universidade de Lisboa, Av. Rovisco Pais, 1049-001 Lisboa, Portugal

[‡]Department of Biomedical Metabolic and Neural Sciences, University of Modena and Reggio Emilia, via G. Campi 287, Modena, 41125 Italy

[¶]Department of Bioengineering and iBB- Institute of Bioengineering and Biosciences, Instituto Superior Técnico, Universidade de Lisboa, Av. Rovisco Pais, 1049-001 Lisboa, Portugal

[§]Associate Laboratory i4HB—Institute for Health and Bioeconomy, Instituto Superior Técnico, Universidade de Lisboa, Av. Rovisco Pais, 1049-001 Lisboa, Portugal

[‖]Department of Bioengineering, Instituto Superior Técnico, Universidade de Lisboa, Av. Rovisco Pais, 1049-001 Lisboa, Portugal

E-mail: ana.pires@lx.it.pt

The PDF file includes:

Figs. S1 to S8

Other Supplementary Materials for this manuscript include the following:

Movies S1 to S2

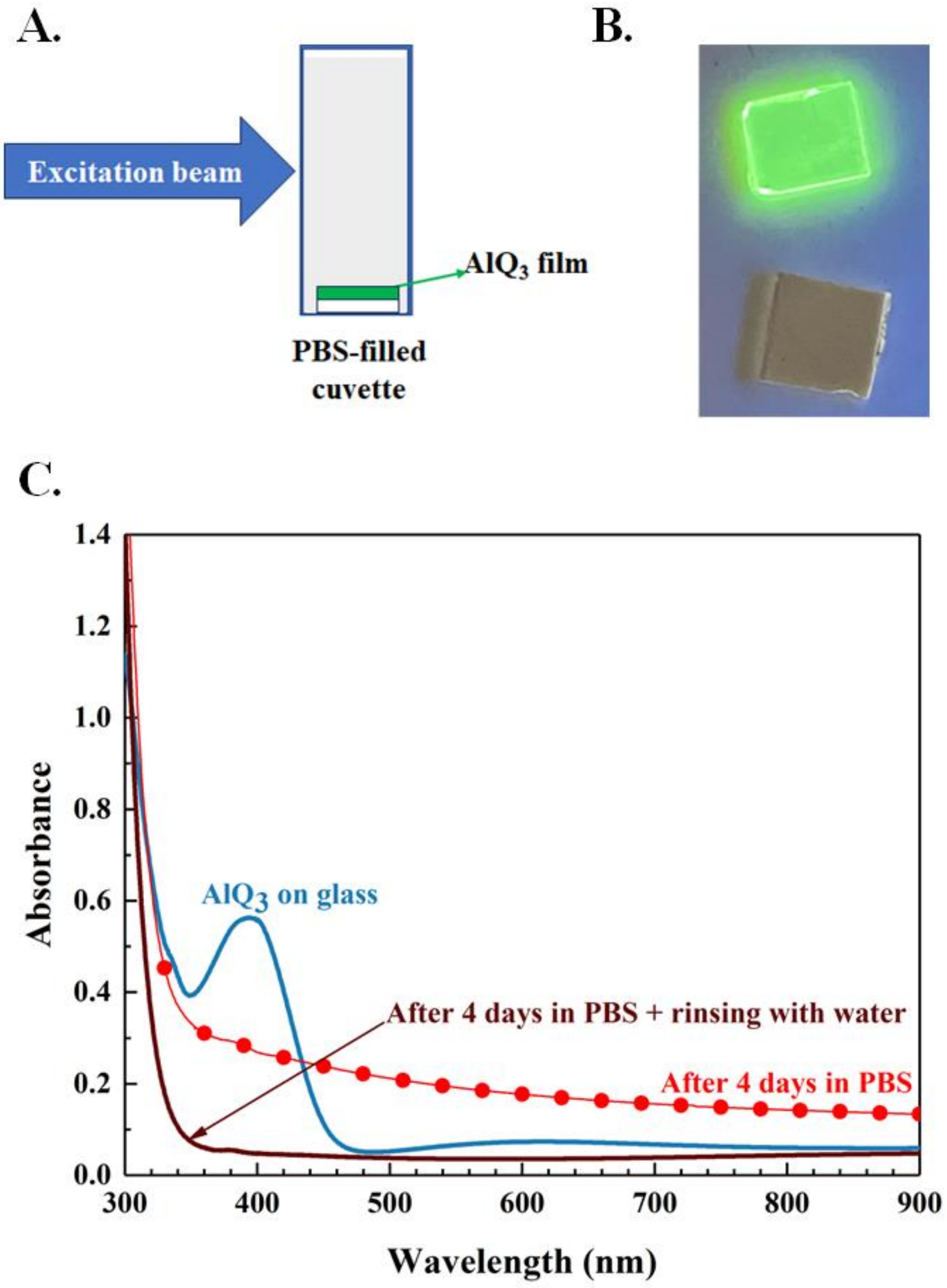

Figure S1. A) Setup scheme used to follow the degradation of the AlQ_3 film upon contact with PBS. The film is positioned in such a way that it is out of the excitation beam trajectory. B) Photos of a 140 nm thick film of AlQ_3 on glass and of the glass that remained after the experiment of Figure S1A, under illumination at 254 nm. C) Absorption spectra of a film of AlQ_3 on glass, after being immersed in PBS for 4 days and then after rinsing with water (release of some degradation products that remained precipitated on the glass substrate).

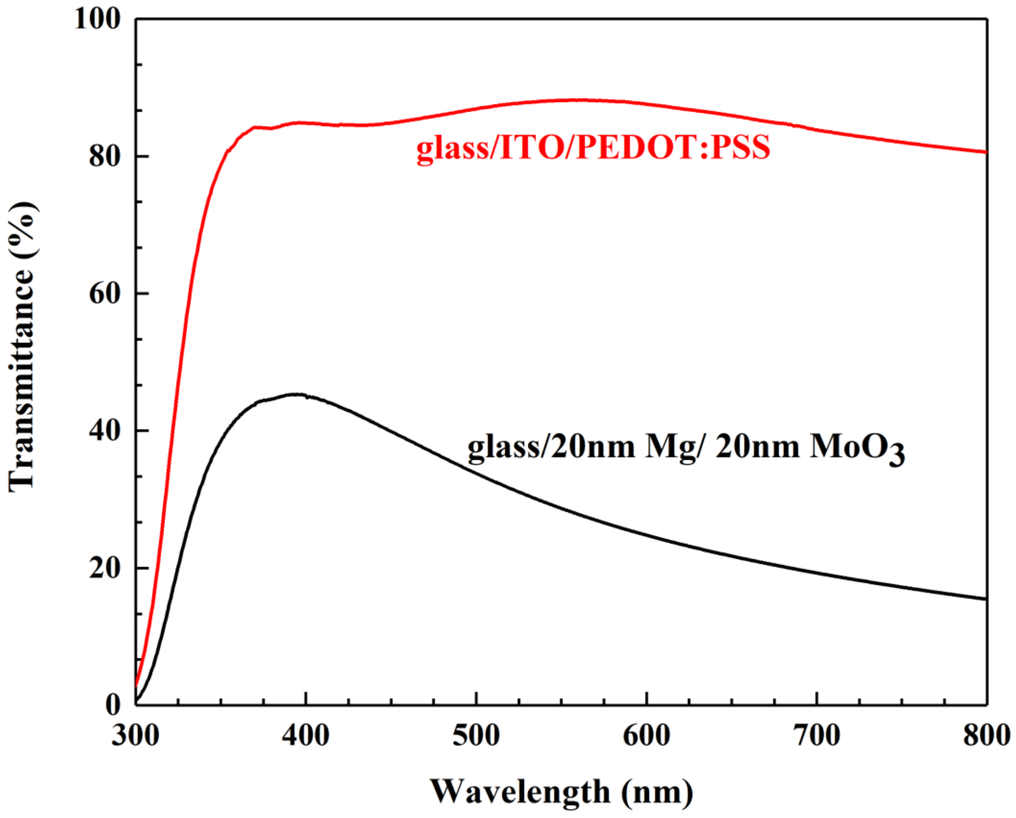

Figure S2. Transmittance of ITO/PEDOT (red line) vs Mg/MoO_3 (black line) both on glass substrates.

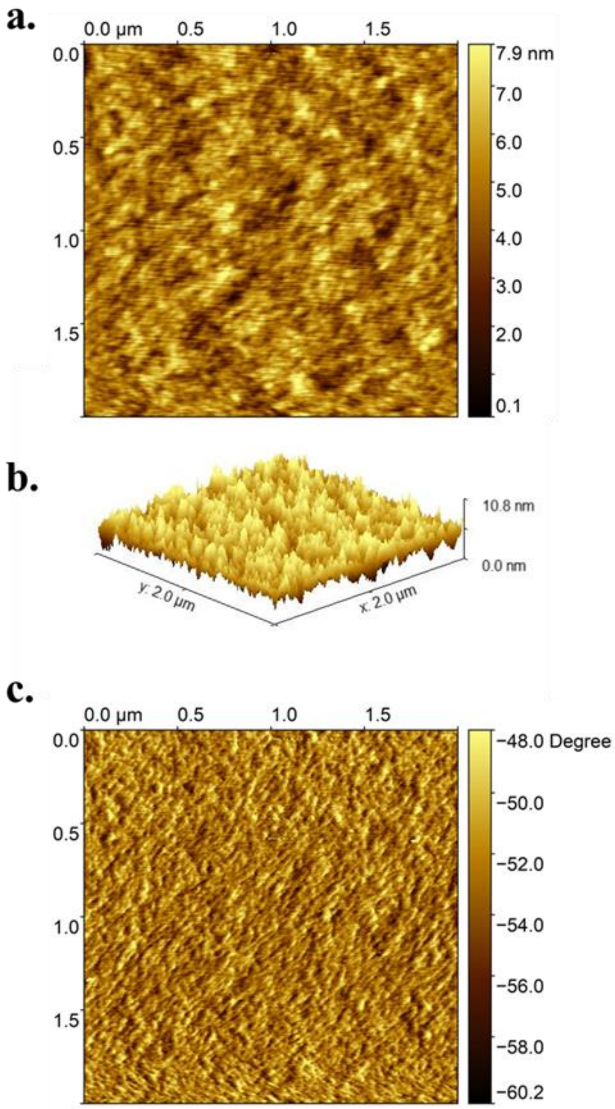

Figure S3. AFM 2D and 3D topography (a, b) images and corresponding phase image (c) of chitosan substrate (scan area is 2 µm × 2 µm).

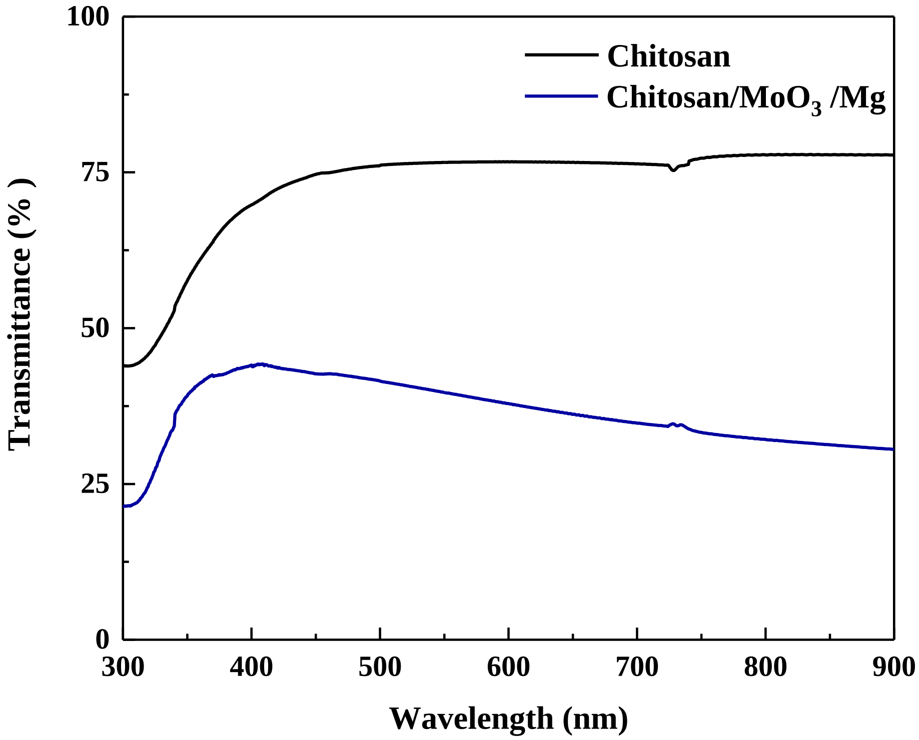

Figure S4. UV–visible transmission spectrum of a chitosan substrate before (black line) and after (blue line) magnesium-based anode formation.

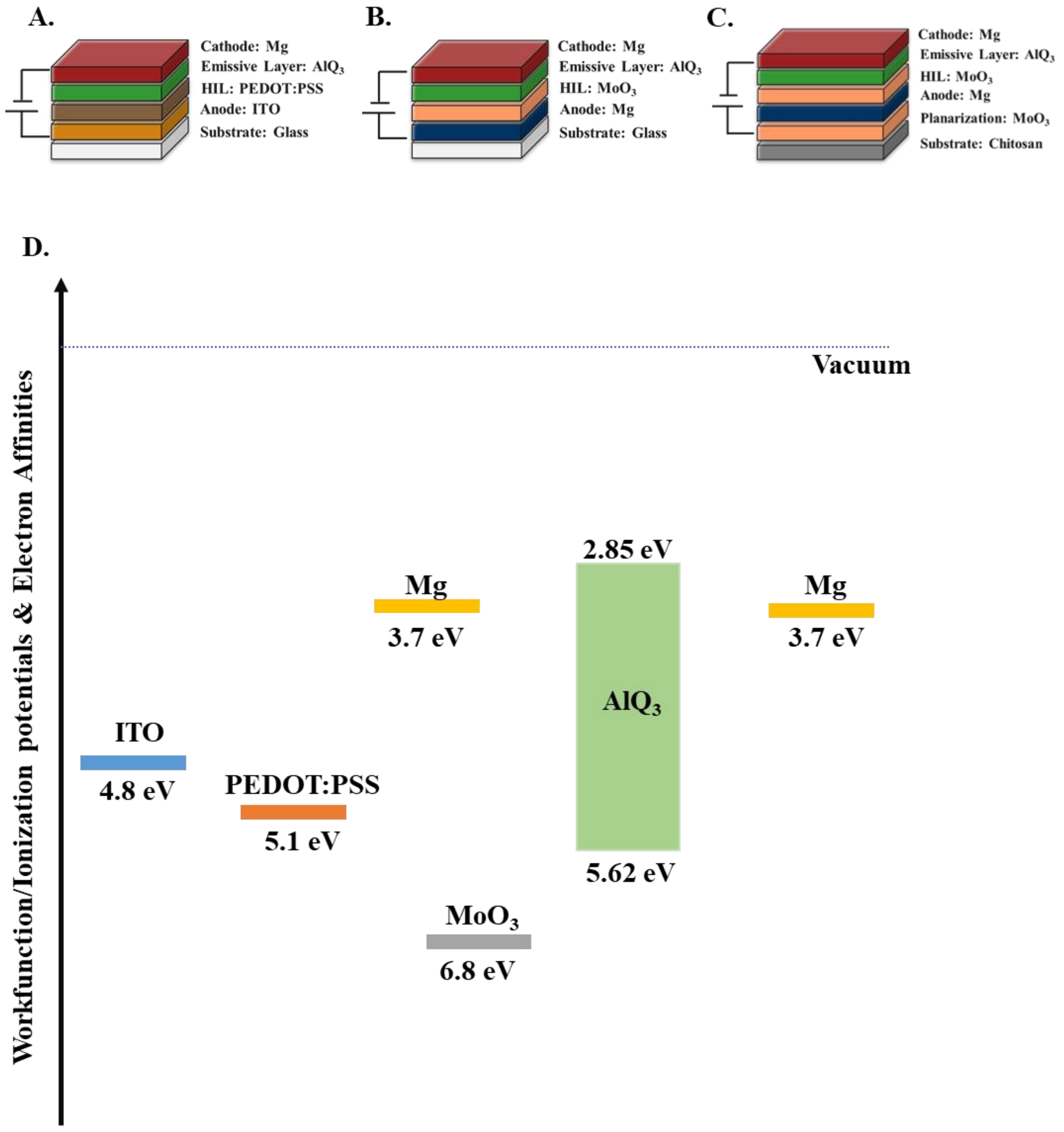

Figure S5. Structure of the OLEDs (A to C) and energy level diagram of the various components (D).

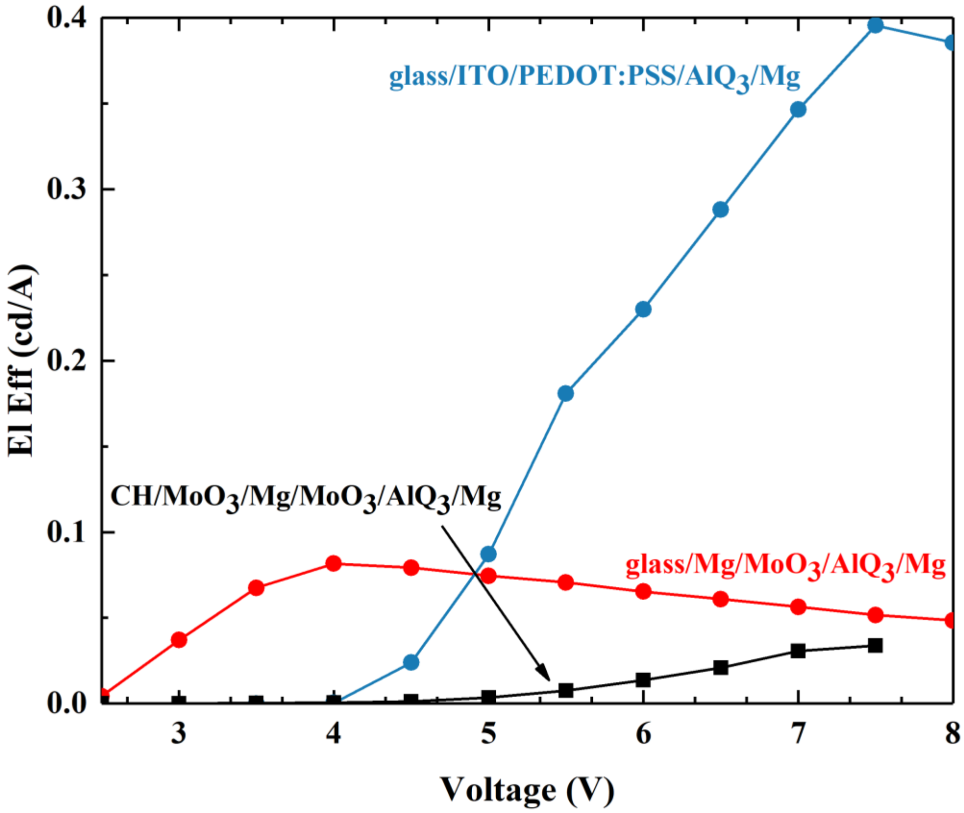

Figure S6. EL eff of the LEDs whose characteristics are shown in Figure 4.

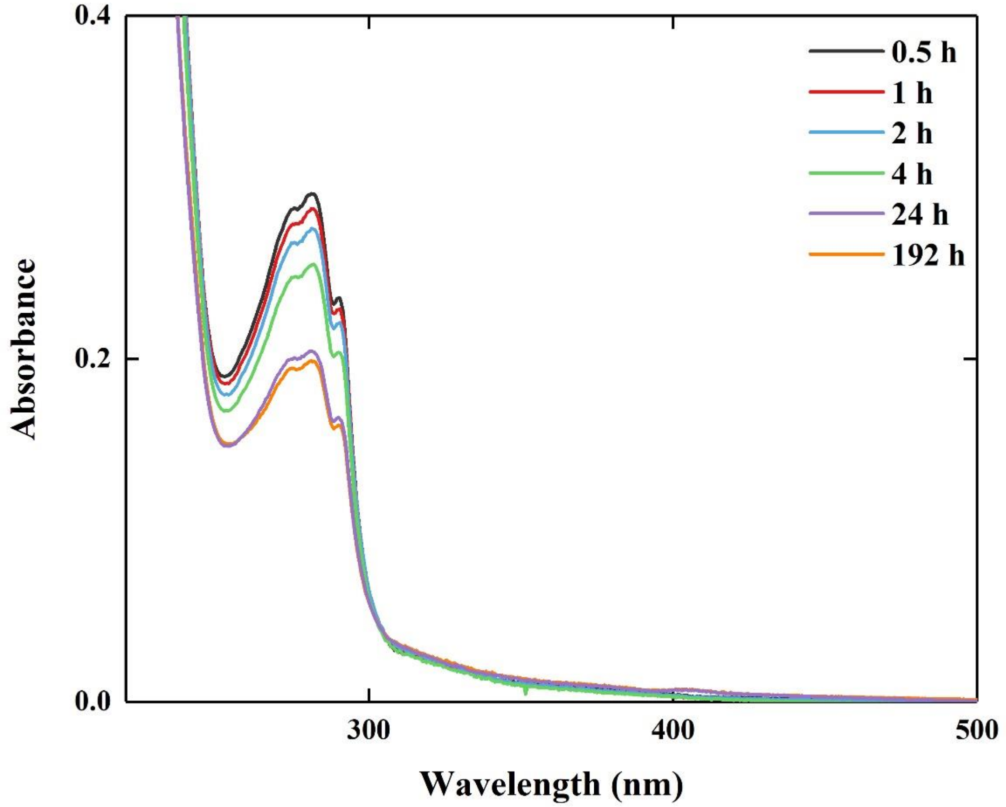

Figure S7. Degradation of chitosan-based OLED immersed in PBS + 1mg/mL lysozyme solution. Absorbance measurements on the PBS + lysozyme solution reveal a decrease in the intensity of characteristic lysozyme bands, indicating its active role in degrading the chitosan-based OLED over time.

a. **b.**

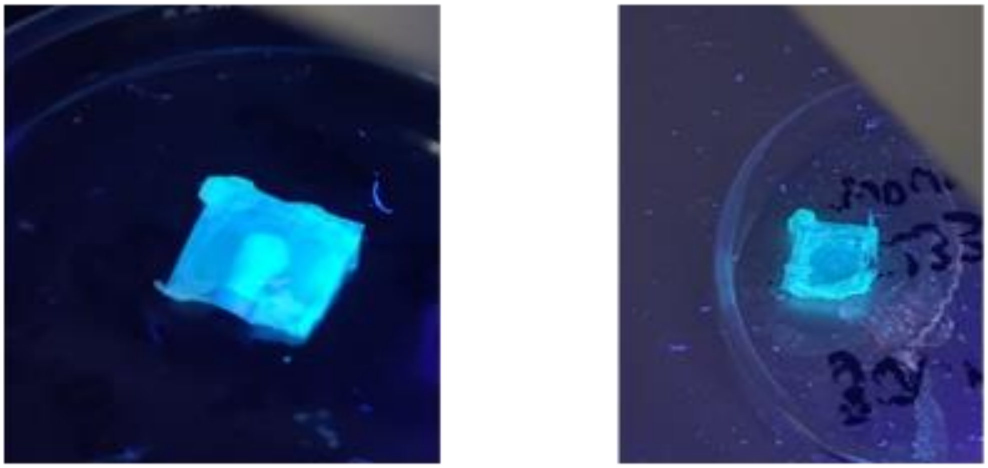

Figure S8. PLGA encapsulation of the chitosan-based OLED (a) improves the barrier against both oxygen and moisture, enabling the magnesium-based electrodes to remain stable for up to five months when stored outside of a glove box (b).

Movie S1.

Movie S1. After 24h of immersion, the degradation of chitosan-based OLED in PBS is limited, as the polymer curled and exhibited poor dissolution.

Movie S2.

Movie S2. In the presence of lysozyme, the chitosan-based OLED undergoes complete dissolution within 24 hours, driven by the enzymatic hydrolysis.
